# LEARNHIV: development and validation of a mobile application for primary health care nurses focused on HIV care/prevention

**DOI:** 10.1186/s12912-023-01579-0

**Published:** 2023-11-09

**Authors:** Melyane de Assunção Gaia, Eliã Pinheiro Botelho, Glenda Roberta Oliveira Naiff Ferreira, Cintia Yolette Urbano Pauxis Aben-Athar, Andressa Tavares Parente, Fabianne de Jesus Dias de Sousa, Marielna Silva dos Santos, Natalia Maria Vieira Pereira-Caldeira, Richardson Augusto Rosendo da Silva, Aline Maria Pereira Cruz Ramos

**Affiliations:** 1 Graduate Program in Nursing, Federal University of Para, Belem, 66075-110 Brazil; 2 Nursing School, Federal University of Para, Belem, 66075-110 Brazil; 3 Department of Maternal and Child Nursing and Public Health, Nursing School, Federal University of Juiz de Fora, Juiz de Fora, Brazil; 4 Postgraduate Program in Nursing, Federal University of Rio Grande do Norte, Natal, Brazil

**Keywords:** HIV, Primary Health Care, Nurse, Mobile Apps, Educational Technology

## Abstract

**Introduction:**

Human immunodeficiency virus (HIV) infection is a relevant public health problem is worldwide. From the change in the health care of people living with HIV (PLHIV) in Primary Health Care (PHC), nurses gained autonomy in their workflow, which requires a significant technological arsenal for the planning, organization and functioning of services. It is believed that the development of a mobile application for the care/prevention of HIV will contribute to the strengthening of care, resulting in greater autonomy and empowerment of nurses in Primary Health Care.

**Objective:**

To develop and validate a content script for a mobile application for nurses in PHC containing information about PLHIV management/care in PHC.

**Methods:**

This is a methodological study developed in three phases: exploratory study, content elaboration process and validation by the 16 judges.

**Results:**

The application was evaluated and validated satisfactorily in terms of content and appearance, with an average Content Validity Index (CVI) of 0.99 (99%), Item Content Validity Index (I-CVI) and Medium Content Validity (S-IVC/AVE) also obtained satisfactory levels.

**Conclusions:**

The construction of the prototype of an application called LearnHIV, is considered a valid instrument in terms of content and appearance, according to the judges.

**Trial registration:**

None because it is not an intervention study.

## Introduction

Human immunodeficiency virus (HIV) infection is a global public health problem, with approximately 39 million people living with the infection in 2022 [1]. In Brazil, according to the report for the year 2023, referring to the period from 1980 to 2022, 1,088,536 cases of infection by the virus were reported, with the highest concentration of cases in the Southeast region with 43.3%, followed by the Northeast region with 19 0.8%, South with 19.7%, North with 9.5%. and Central-West with 7.7% [2, 3]. The treatment against HIV is provided free of charge by the Unifed Health System (SUS, in portuguese), it is an example of a successful model for the entire world [4]. Despite these advances, the current data indicate that the temporal trend was decreasing in Brazil and all regions, except the North and Northeast [3]. The North Region showed a growth in the incidence rate in the general and younger age groups up to 29 years old related to sociocultural conditions and greater vulnerability to infection, morbidity, and mortality [3], as well as limitations on access to healthcare or active case finding and adherence to treatment [5].

In 2000, UN member countries developed eight Millennium Development Goals (MDGs), which included targets to be met between 2000 and 2015, among which, target 3 “Health and Well-Being” stands out, specifically the subitem 3.3 “By 2030, end the epidemics of AIDS, tuberculosis, malaria and neglected tropical diseases, and combat hepatitis, waterborne diseases, and other communicable diseases” [6].

Primary Health Care (PHC) is the gateway to all levels of healthcare [7] but to HIV its should improve to HIV prevention and its treatment. Since 1996, the Ministry of Health has distributed monthly antiretrovirals monthly and in 2012 rapid testing began in PHC to detect HIV [8]. In 2014, there was a reorganization of the care model for people living with HIV (PLHIV), prioritizing primary care as the gateway and organizer of care. Through this model, we sought to increase accessibility to health services for these users [9].

The city of Florianopolis (southeastern Brazilian) showed positive results in decentralization about regarding access enlargement and qualification of care for people living with HIV (PLHIV) [10]. However, other regions have challenges such as lack of care protocols capable of providing specific guidance to professionals [9], mainly, the nursing staff [10].

Nursing is the largest category of PHC, developing its role in a relevant way in Basic Health Units (BHU) with Family Health Strategy (FHS) to consolidate the HUS model in SUS. Recently, it has been observed that nurses who base their work on authentic health information are able to make clinical decisions and provide higher quality care in this model [11], but this workforce needs to be more directed towards HIV prevention and management [12]. The HIV patients care, and prevention is complex and requires numerous skills and attention according to their particularities, which requires some nurses to be constantly up to date. Nurses must gather specific skills and abilities to encourage adherence to preventive measures, testing, and follow-up of patients with the infection without discrimination among heterosexuals and key populations [13]. In addition, this complexity increases in Amazonian due to its heterogeneous geographical, climatic and population traditional (Indians, Quilombolas, riverside dwellers and others) whose nurses need to overcome these specificities and strengthen ties to provide care [14]. With the advancement of technology, mobile health app and other digital health interventions seem to have come to the fore in recent years in the face of facilitated accessibility [15, 16]. Emerging through the concept forefront mobile health (mHealth), as an application of mobile technology with the aim of improving health outcomes through the transmission of health data and the acquisition of health goal information. Smartphones have become tools widely used as a method of obtaining health information quickly and easily, however, it has become clear that there is no application aimed at the practice of nursing care for patients [14].

There are few applications aimed at the care provided by nurses. The PEPtec app [17] was developed to assist health professionals in assessing the risks to which users were exposed to HIV, to support decision-making regarding PEP recommendations. While the development of the app “Occupational exposure to HIV – EoHIV” [18], aims to encourage health professionals to adhere to prophylactic treatment with antiretrovirals indicated after occupational exposure to potentially contaminated biological material.

However, no application brought the integral approach of the patient in HIV by the nurse. Thus, the insertion of an app in the health area comprehensive approach can be understood as an ally to professionals, however, there was no evidence of an app aimed at nurses, thus consequently emerging the potential of the developed yet app in vogue.

Therefore, this study aims to develop and validate the design and content of a mobile application for PHC nurses on the management and care of people living with HIV.

## Methods

### Study design

**Fig. 1:**
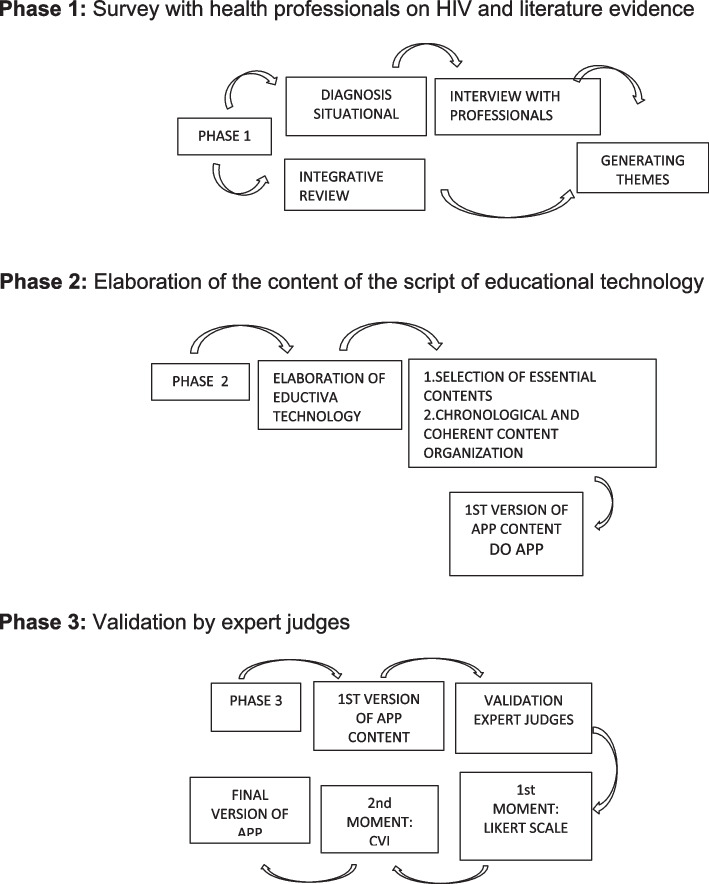
Phases of the study

This is a methodological study on the production, evaluation, validation, and improvement of the content of a script for a mobile app as a strategy for the prevention and management of PLHIV in PHC. The study was conducted in three phases (Fig. 1).

### Study design

#### Phase 1

In the initial phase, a situational diagnosis was carried out to assess knowledge about HIV care/prevention to identify the main weaknesses and strengths in the work practices of nurses working in PHC in 6 peripheral and populous neighborhoods of the city of Belem, in the state of Para, Brazil. The total population of these neighborhoods adds up to 309,223 people [16] who are assisted by 6 ESFs with 35 nurses. It is known that each FHS must serve up to 4,000 people in the territorial region under its responsibility, but there is a shortage of professionals and coverage is not ideal, with coverage of 22% in the state of Para [17].

For the calculation of the sample of the situational diagnosis, a finite population of 35 nurses, a 95% confidence interval and a 5% margin of error were considered, and it was performed in SPSS v.24. A sample of 33 professionals was reached, however, all were approached and 3 (5.71%) did not agree to participate.

A validated questionnaire was used and adapted to evaluate HIV control actions in primary care [19], which served as a parameter for the thematic base of the mobile application. 33 nurses working in PHC participated in this stage, who signed the Free and Informed Consent Form (TCLE).

Then, Microsoft Excel for Windows® version 2016 software was used to double-check and correct inconsistencies. Subsequently, the statistical software IBM® Statistical Package for the Social Sciences version 20.0 was used, which helped in the descriptive analysis of the situational diagnosis report. Another parameter used as thematic base for the mobile application was the literature review about the knowledge relevant to the performance of nurses related to HIV in PHC in the main databases, such as PubMed, Scielo, BVS, and Medline. “HIV” with the Boolean operator “OR” “Primary Health Care” was used as DeCS/MeSH descriptors, associating them with the operator “AND” and the descriptor “Acquired Immunodeficiency Syndrome” and “Nursing.” The inclusion criteria applied referred to studies published in up to 5 years in the languages: English, Spanish, and Portuguese on the theme of HIV in PHC and were available in full and free of charge.

#### Phase 2

In phase 2, the application content development process was carried out, supported by the technical-scientific contents selected from the RIL, the evidence of the situational diagnosis. Then, this process was added to the Clinical Protocol of Guidelines for people with sexually transmitted infections (STIs) [20].

#### Phase 3

In phase 3, for apparent and content validation of the prototype, two groups of expert judges were used: from the health area and from other areas. Each group of judges was responsible for carrying out a type of validation that contributed to the optimization of the technology, with this population being made up of health professionals, teachers and professionals who work in assistance, with expertise in the topic addressed by the software, in addition to professionals in the field. of education. Those in the health area were 13 nurses selected from a curricular search on the Lattes Platform, available on the Portal of the National Council for Scientific and Technological Development – CNPQ. The defined inclusion criteria were knowledge of Sexually Transmitted Infections (STIs), experience in caring for people diagnosed with HIV in public or private services. The judges from the other areas were responsible for the didactic-illustrative validation of the app prototype, selected after consulting the Lattes curriculum, which stipulated: a professional Pedagogue, a social communicator, and a graphic designer, who had specialization in their professional area and two years of experience, following Fehring's model of inclusion criteria [21] and those individuals who reached at least five points were selected, as recommended by the adopted framework.

After selection, an invitation letter was sent via email (personal and institutional) or through the “contact” section of the Lattes Platform, with a description of the objectives, purposes of the study and identification of researchers (personal and institutional). After confirming participation, the professional received the consent form and the content of the prototype in PDF format. In addition, a link was sent to access the evaluation instruments in electronic format, establishing a maximum period of 05 (five) days for the subsequent phases of the research to continue.

The application's apparent and content validation process was carried out by expert judges (nurses and other areas) based on the evaluation of the scientific and technical content and organization of information in accordance with the Clinical Protocol and Guidelines for Comprehensive Care for People with STIs [20].

For this, a content, appearance, and didactic-illustrative validation instrument was used by the expert judges, containing 18 objective questions with four answer options organized on a Likert scale: 1 “totally disagree”, 2 “somewhat disagree”, 3 “I somewhat agree” and 4 “totally agree”, and the last stage consisted of adapting the final version of the application’s content, based on the suggestions of the judges from both areas.

It should be noted that items that did not reach the minimum agreement value of 0.80 were reviewed [21]. The results were analyzed globally and by item, using the Content Validity Index (CVI), Content Validity Index per item (IVC-I), which validated the items individually; Average Content Validity Index (S-IVC-AVE), which each expert judge evaluates as relevant and very relevant. Items with a minimum value of 0.80 were validated, those that did not reach this score were revised [22].

**Fig. 2:**
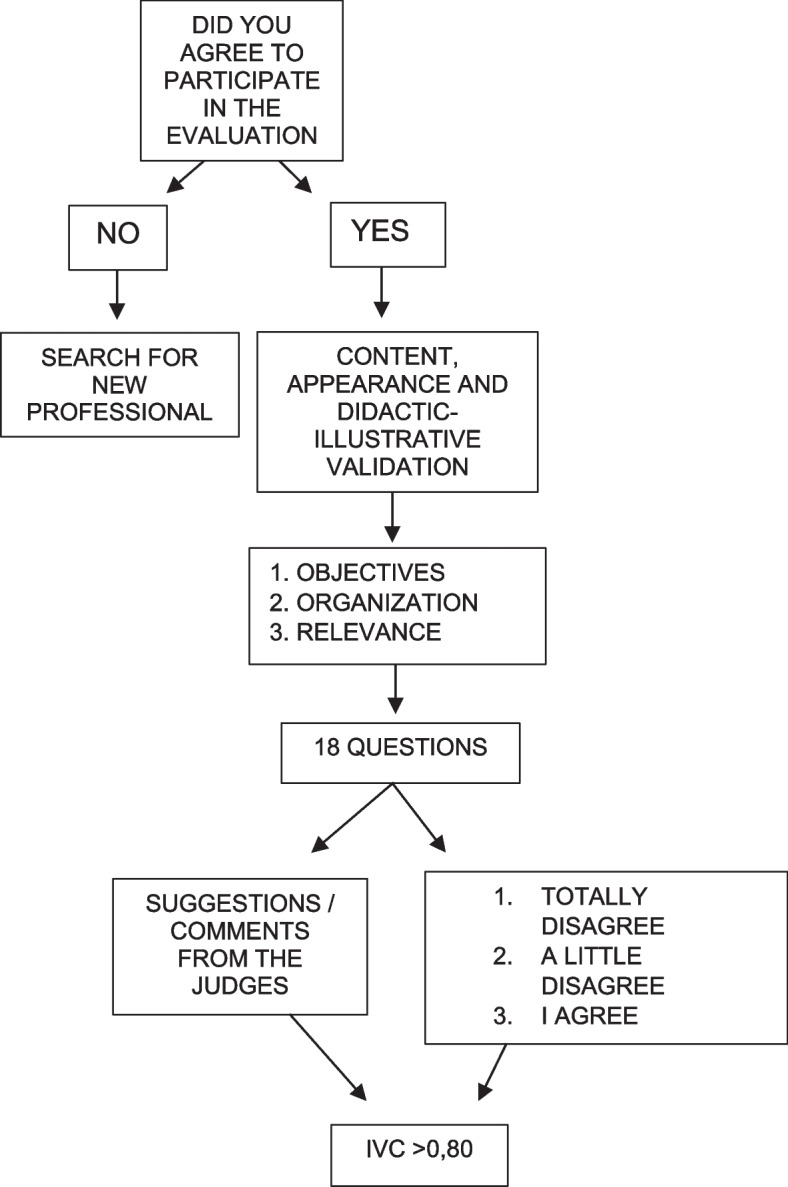
Decision tree for phase 3 in the validation process. Belem, Para, Brazil, 2022

The instrument evaluated items related to evaluation scores from 1 to 4, which addressed: 1. Objectives; 2. Organization; 3. Relevance. It should be noted that below each block of questions a space was made available for comments and/or suggestions from the expert judges Fig. 2.

As for the calculation of CVI, the following formula was used:

$$\frac{\mathrm{IVC}=\mathrm{total\,number\,of\,answers\,}\,3\,\mathrm{or}\,4}{\mathrm{total\,number\,of\,answers}}$$

The instrument used in this step was a questionnaire for evaluation objectives, organization and relevance built in Google Forms based on research by Nascimento (2012) [23] and adapted for this study, in which the Likert scale was applied to define the degree of agreement of the expert judges. After being sent to the judges, the data were exported to a Microsoft Office Excel 2010 spreadsheet (Microsoft Corporation; Redmond, WA, USA). Statistical analyzes were performed using the SPSS ® version 20 ® program and presented in the form of graphs and tables.

### Ethics and consent

The study was approved by the Research Ethics Committee of the Federal University of Para under protocol number 3,488,663 and followed all the principles of the Declaration of Helsinki AND complied the with the Regulatory Guidelines and Norms for Research Involving Human Beings, in accordance with the recommendations of Resolution 466/2012, 510/2016 and 580/2018 of the National Health Council. All study participants signed an informed consent form. None of the participants received rewards for participating in the study.

## Results

### Profile of APS Nurses

**Fig. 3:**
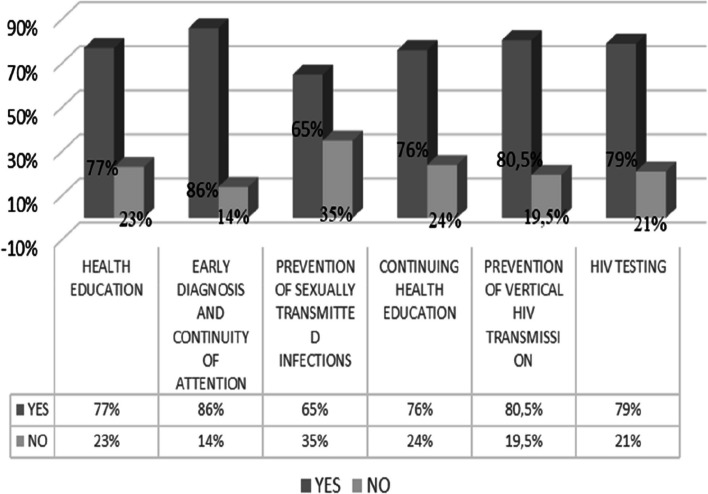
Domains with weaknesses identified in professionals

In the first phase, 33 Brazilian professionals were interviewed. Almost all are women, with an average age of 40.8 years, average monthly income between 2.9 minimum wages, average of 12.4 years oldof professional experience in PHC, Catholic (68.7%) and married (51%). From the adapted instrument, it was possible to verify, through six domains, which were the areas of greatest fragility, with emphasis on the domains related to the prevention of STIs (35%) and the need for permanent health education (24%) as shown in Fig. 3.

### Integrative literature review

In the first phase, RIL was carried out in June 2021, the nurse faces several challenges peculiar to its daily practice, mainly related to the routine of professionals in the PHC, ranging from the execution of the test for the diagnosis of HIV (testing) until the moment of the positive result, in addition to the deficit of material inputs, demystification of the stigma and prejudice suffered by users, as well as gaps in training offered to professionals. However, it should also be considered that the APS emerges as a space that enhances knowledge in the sense of improvement and accessibility, especially about early diagnosis for HIV detection.

Since it is imperative to expand the training of nurses working in PHC, enabling training for difficult communication in health, which is understood as a mechanism to assist these professionals and prepare them to support, empower and make the user co-responsible for their treatment. It is important to emphasize the relationship interprofessional and collaborative, where the other members of the multidisciplinary team can act more strongly also in accountability for the diagnosis, adherence, treatment and monitoring and support for this user, further strengthening egalitarian care, resolute and comprehensive.

### Creating the application layout

The second phase comprised the organization of content into themes and, subsequently, the condensation of information from previous phases based on official recommendations and legislation from the Federal Nursing Council, instituted in accordance with Law 5.905/1973 (responsible for regulating and supervising compliance with the Law of Professional Nursing Practice) [24]. This case regulates the administration of rapid tests and the technical skills involved in each category. Based on this information, the following application menu tabs were created: “Combination prevention mandala”, “Prevention of HIV Transmission”, “Early Diagnosis”, “Permanent Health Education”, “Test your knowledge” and “Points of Attention on the Network.”

In the creation of the layout, still in phase two, design thinking [23] was defined, which was initially built based on research into the main functionalities in mobile applications used in the health area and already available in the market for Android and iOS Operating Systems.

Ease of reading, clarity of information, attractive appearance, and good usability were also considered, easily read using the same Arial Narrow font, size 11 for titles and size 9 for texts, practicality on all tabs. The colors used are sought to be pleasing to the eye, harmonic, with good legibility in communication and to emphasize the theme of HIV. White and red were then defined as comprehensive colors for the prototype. The colors white and red were defined as predominant due to the HIV theme and for the main menu of the app, the other colors used are related to key population [25, 26].

The choice of infographic elements was defined to cover the themes addressed in the topics, the target audience, and the Amazonian population. All icons used in the application’s layout were created by a designer and released for use, free from copyright.

### Validation of application content and appearance

In the third phase, 13 nurses participated in the validation as expert judges, 11 females and two males. Among them, six are from the Southeast region, five are from the North, and two are from the Northeast region. Moreover, two are post-doctors, and 11 are doctors, with 38.5% of the judges working as professors or researchers in Transmissible Diseases in Public Health. Table 1 shows the judges’ assessment of content and appearance.

**Table 1:** Distribution of scores and percentage of consensus based on expert judges’ assessment of each item, according to the objectives, organization, and relevance in the 1st and 2nd round. Belem, PA, Brazil, 2022

Evaluation								
Appearance and Content Category	**N**	DT*	DP*	CP*	CT*	**1° IVC**	**2° IVC**	**SCORE**
		1	2	3	4	0,80	0,80	
Block 1—Objectives								
**1.1**	16	0	0	2	14	0,93	1,00	+ 1
**1.2**	16	0	0	1	15	0,93	1,00	+ 1
**1.3**	16	0	0	0	16	0,92	1,00	+ 1
**1.4**	16	0	0	0	16	0,93	1,00	+ 1
**1.5**	16	0	0	2	14	0,93	1,00	+ 1
**Subtotal**	80	0	0	5	75	0,92	1,00	
**Percentage by value**				6,25%	93,7%			
Block 2—Organization								
**2.1**	16	0	0	1	15	0,93	1,00	+ 1
**2.2**	16	0	0	3	13	0,93	1,00	+ 1
**2.3**	16	0	0	1	15	0,81	1,00	+ 1
**2.4**	16	0	0	1	15	0,81	1,00	+ 1
**2.5**	16	0	0	3	13	0,87	1,00	+ 1
**2.6**	16	0	0	2	14	1,00	1,00	+ 1
**2.7**	16	0	1	1	14	1,00	0,93	+ 1
**2.8**	16	0	1	0	15	0,87	0,93	+ 1
**Subtotal**	128	0	2	12	114	0,90	0,98	
**Percentage by value**				9,37%	89%			
Block 3- Relevance								
**3.1**	16	0	0	0	16	0,93	1,00	+ 1
**3.2**	16	0	0	2	14	0,87	1,00	+ 1
**3.3**	16	0	0	1	15	0,87	1,00	+ 1
**3.4**	16	0	0	6	10	0,81	1,00	+ 1
**3.5**	16	0	0	1	15	0,93	1,00	+ 1
**Subtotal**	80	0	0	10	70	0,89	1,00	
**Percentage by value**				12,5%	87,5%			

Legend: *DT *I totally disagree, *DP *Partially Disagree, *CP *Partially Agree, *CT *I totally agree, *CVI *Concordance Validity Index

Source: Authors (2022)

The IVC-AVE of all items was greater than 0.80, both for the objectives, organization, and relevance, indicating that the application presented satisfactory evaluation, reaching an overall mean value of 0.99 (99%). In the evaluation items, there was a variation from 0.07 to 0.90. It is emphasized that the judges' suggestions were analyzed, incorporated into educational technology, and later the instrument was reevaluated by the judges, according to Table 1.

In general, after calculating the total CVI scores, in the two validation rounds, from each judge, a variation between 0.92 and 1.00 of absolute agreement was observed, with an average of 91%, indicating a high level of agreement (greater than 90%). And of the 16 responding judges, 14 (87.5%) completely agreed with all items in the questionnaire. Thus, it appears that the content of the mobile application was evaluated positively Table 2.

**Table 2:** Summary of the analysis of the changes suggested by the expert judges. Belem, PA, Brazil, 2022

Item	Block 3—Relevance	
	Suggestions and comments from the judges	Suggested and accepted modifications
**J.2**	I suggest adding information on where to refer a pregnant woman who tested positive for HIV, a normal person, the need to actively search for partners, the need for CD4 t cells monitoring, viral load, weight, and others	Build a tab on the menu that helps professionals guide the user to the correct location, making their itinerary as short and effective as possible
**J.5**	The contents of the interfaces do not follow a script. I suggest removing the QUIS and keeping only the HIV information. In the interface on mother-to-child transmission, you address only HIV, HIV is an STI, but your approach seems not to be about STIs	Keep the focus of the content on information relevant to HIV, avoiding inserting too many STIs Align the content script so that it is logical and intuitive
**J.6**	Greater emphasis is needed on risk assessment. This item was little treated and is very important for health promotion and disease prevention (STI)	Approach in more detail the risk assessment for HIV infection, offering professionals the necessary subsidies for their daily practice

Legend: J.1: Judge1 J.2: Judge 2; J.3: Judge 3; J.4: Judge 4; J.5: Judge 5; J.6: Judge 6.

Source: Authors (2022)

Then, the global evaluation, the I-CVI, was carried out, where the number of judges who partially and totally agreed with the statements was evaluated. It was divided by the total number of judges and IVC-Ave, where the number of questions was evaluated based on the totally and partially agreed answers to the total number of questions. Notably, in both moments, there was an improvement in the quality and consistency of the information, as presented in Table 3.

**Table 3:** Results of the I-CVI and IVC-Ave calculations after the second round referring to the items in the blocks—objectives, organization, and relevance

QUESTION ASSESSED	J1	J2	J3	J4	J5	J6	J7	J8	J9	J10	J11	J12	J13	J14	J15	J16	I-CVI
**BLOCK 1 – OBJECTIVES**																	
** Q1.1**	4	4	4	3	4	3	4	4	4	4	4	4	4	4	4	3	1,00
** Q1.2**	4	4	4	4	4	4	4	4	4	4	3	4	4	4	3	4	1,00
** Q1.3**	4	4	4	4	4	4	4	4	4	4	4	4	4	4	4	4	1,00
** Q1.4**	4	4	4	4	4	4	4	4	4	4	4	4	4	4	4	4	1,00
** Q.15**	4	4	3	3	4	4	4	4	3	4	4	4	4	4	4	4	1,00
** S-CVI/AVE**	1,00	1,00	1,00	1,00	1,00	1,00	1,00	1,00	1,00	1,00	1,00	1,00	1,00	1,00	1,00	1,00	-
**BLOCK 2 – ORGANIZATION**																	
** Q2.1**	4	4	4	3	4	4	4	4	3	4	4	4	4	3	4	4	1,00
** Q2.2**	4	3	4	3	4	4	4	4	4	4	4	4	4	4	4	4	1,00
** Q2.3**	4	4	4	3	4	4	4	4	4	4	4	4	3	4	4	4	1,00
** Q2.4**	4	4	4	3	4	4	4	4	4	4	4	3	4	43	4	4	1,00
** Q2.5**	3	4	4	4	4	4	4	3	4	4	3	4	4	4	4	4	1,00
** Q2.6**	4	3	3	3	4	4	4	4	4	4	4	4	4	4	4	4	1,00
** Q2.7**	4	4	4	4	4	4	4	4	4	4	4	4	4	4	4	4	1,00
** Q2.8**	4	4	4	4	4	4	4	4	4	4	3	3	4	4	4	4	1,00
** S-CVI/AVE**	1,00	1,00	1,00	1,00	1,00	1,00	1,00	1,00	1,00	1,00	1,00	1,00	1,00	1,00	1,00	1,00	-
**BLOCK 3 – RELEVANCE**																	
** Q3.1**	4	4	4	4	4	4	4	4	3	4	3	3	4	4	4	4	1,00
** Q3.2**	4	4	4	4	4	4	4	4	4	4	4	4	4	4	3	4	1,00
** Q3.3**	4	4	4	4	4	4	4	4	4	4	4	4	4	3	4	4	1,00
** Q3.4**	4	4	4	3	4	4	4	4	3	4	4	4	4	4	4	4	1,00
** Q3.5**	4	4	4	4	4	4	4	4	4	4	4	4	4	4	4	4	1,00
** S-CVI/AVE**	1,00	1,00	1,00	1,00	1,00	1,00	1,00	1,00	1,00	1,00	1,00	1,00	1,00	1,00	1,00	1,00	-

Legend: *I-CVI *Content Validity Index per item, *S-CVI/AVE *Medium Content Validity Index (S-IVC-AVE)

Source: Authors (2022)

**Fig. 4:**
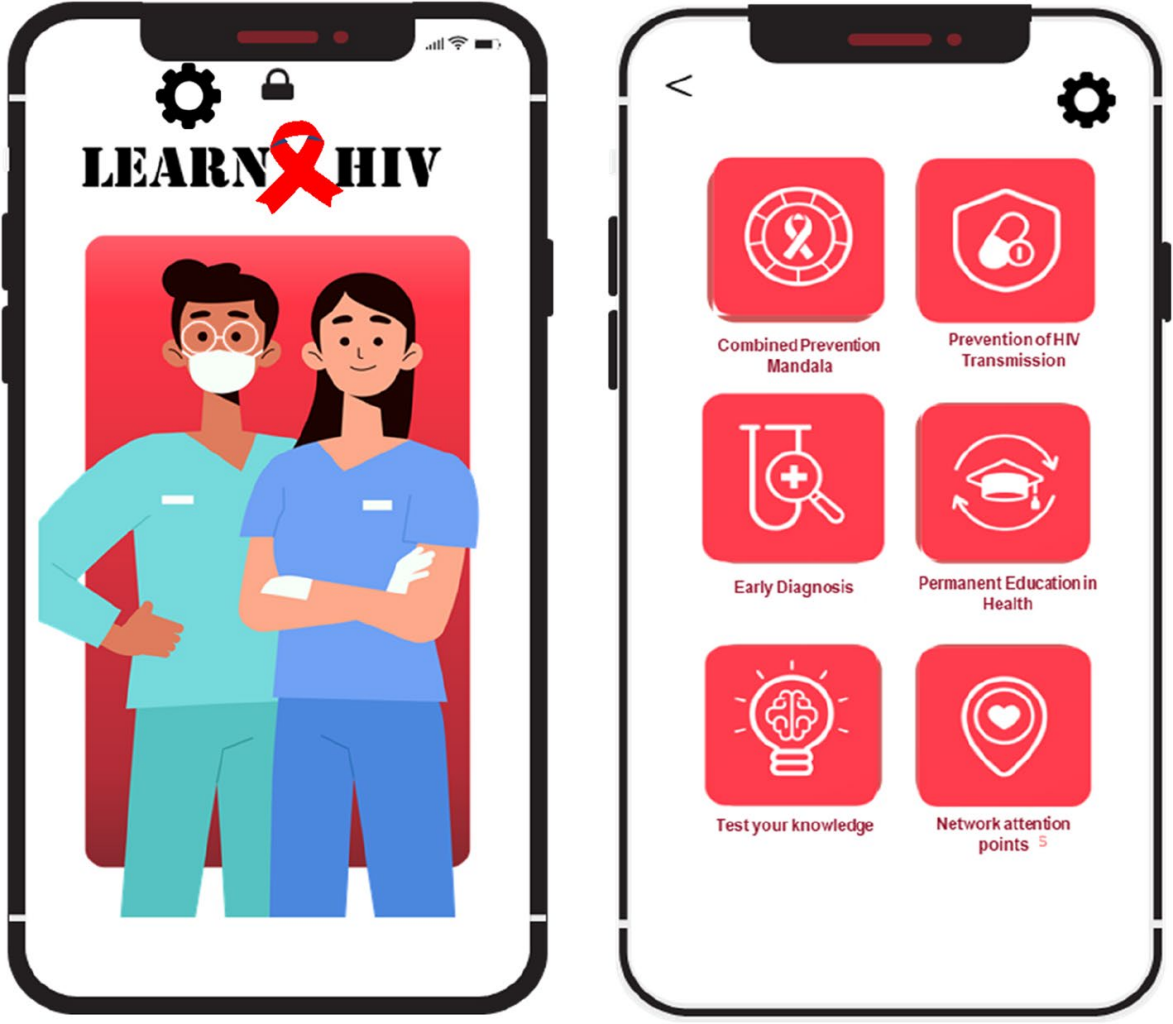
Final version of the application after two rounds of contributions from expert judges. Belem, PA, Brazil, 2022

As for the final version of the application’s layout, the initial screen was defined as the application’s visual identity and later, and the main menu was presented, with the subtabs in Fig. 4.

## Discussion

Identify the knowing the difficulties and limitations of nurses in rhyming health care, as well as the evidence on this topic, was important for the development of the application’s content. The validation process by the expert judges showed that the application’s content is valid regarding the management of HIV, with a global CVI considered excellent. Thus, the application will contribute to professional improvement by clarifying doubts, positively impacting STI prevention actions, including HIV, and signaling significant changes in health promotion that tends to directly reflect on the drop-in HIV infection rates. The development of an application, such as LearnHIV, includes carrying out a rigorous methodological process to guarantee the validity and effectiveness of this application.

In the first phase, through the situational diagnosis, PHC nurses have weaknesses concerning permanent health education and knowledge about STI prevention in the management of PLHIV. This problem has already been evidenced by a previous study [20] carried out in southern Brazil, which showed the need to establish a care flowchart, including the continuing education of professionals. Although the decentralization of testing for PHC was implemented in 2012, another study carried out in 2021 [21] revealed that professional nurses still feel the need for greater qualifications to perform tests and provide care to PLHIV.

Owing to the functions performed by nurses in PHC, they play a fundamental role in the fight against HIV. Thus, technologies that contain easily accessible information in clear and understandable language should be established. However, even after years of decentralization of HIV testing and treatment of PLHIV for PHC, integrative literature review pointed out that there are few technologies developed for nurses [17].

Thus, within phase 2, a difference exists between the app developed in our study and the others regarding the incorporation of information on HIV management from the notebooks and manuals of the Ministry of Health, specifically bringing the role of nurses within the process based on a legal basis. In addition, it features infographics and flowcharts that facilitate professionals’ understanding of actions for the decentralization of care.

In this way, through the app, benefits could be obtained in the fight against HIV, given that it can be used on any mobile device. In addition, according to the reality of the FTS, there are many Brazilian places without computers and Internet access. Moreover, the improvement in professional qualification [23], access to health services, quality improvement, and the resolution of health problems stand out. All these benefits bring improvements in care while facilitating the access of quilombola, riverside, and indigenous populations to PHC care [14].

In step three, which included the application development, an attractive layout was considered, where the information and images were well organized and expressive, giving meaning to the texts. The figures that are part of the app’s content aim to facilitate the user’s understanding and, for that, include characters, scenarios, and experiences closer to the target audience, providing the opportunity to build new meanings and allowing the understanding of everyday life [25]. Application design processes illustrate or need to illustrate the performance of methodological and interpretive preparation in inserting motivational, playful, and attractive content to promote changes in user behavior [26]. The emergence of highly qualified professional nurses allows the use of multiple scientific knowledge while evaluating the items, collaborating for a more robust and critical analysis, with suggestions pertinent during the validation process.

Thus, the use of mobile health applications by nurses is directly linked to health promotion practices, enabling the tracking of indisputably fundamental health indicators for maintaining the health of individuals. As a result, it culminates in important roles in-volving the provision of clinical care, consultations, accurate treatment follow-up and teaching methods of disease prevention [27].

In content validation, the global CVI was 0.99 (99%), indicating that validation is one of the mechanisms for developing effective materials. The reason is that it assesses whether they have achieved their objectives and goals, whether they are accessible and ap-propriate to the public at the time, which it is intended, and whether they are significant for the area of intervention based on their applicability [22]. The I-CVI and S-CVI/AVE also achieved satisfactory levels.

The contribution of the judges who participated in the study was crucial for im-proving the quality of the content, illustrations, and language. During the validation process, the specialists judged the objectives, organization, and relevance of the educational technology prototype, revealing adjustment points, changed, or included, given the continuous improvement of the material presented and confirming the relevance of the study.

Therefore, this technology has innovative potential because it was developed by a nurse and is directed to all professionals, most importantly, nurses. It is fully produced in language appropriate to the level of education, universally, and free of charge. Further-more, the prototype of the application was built to provide nurses working in PHC with an active participation in the care-education process of patients, strengthening their practice of health education and autonomy in health care.

### Limitations

As a limitation of the study, the difficulty in attracting expert judges stands out, since these professionals are often overwhelmed by other demands of their daily routines, not having the flexibility to participate in the validation process, such as the one that occurred in this study. However, the ideal number of judges was reached, allowing the execution of the study.

## Conclusion

At the end of this study, we concluded that the proposed objectives were achieved, therefore, the construction and content validation of the prototype of a mobile phone application entitled “LearnHIV”, has the potential to revolutionize the way HIV is managed by Nurses.

The construction and validation of the LearnHIV content provides valuable information about the health and conduct of professionals when dealing with PLHIV, allowing for an in-depth understanding of the conduct of the infection, as it is synthesized and presented clearly, objectively, organization and direction.

The integration of general information about HIV, ongoing health education and geolocation of basic units for the care of PLHIV, allows us to explore in greater depth the services offered by the network and provide more appropriate and humanized assistance, as the app stands out as a necessary technology suited to information needs on infection prevention, as well as support for decision-making within the scope of Public Health.

In a country of immense cultural and socioeconomic diversity, and with many regions presenting geographic barriers that make it difficult to connect PHC to the long-distance network, LearnHIV is promising to further qualify nursing care for PLHIV and improve surveillance around HIV. Sexuality and population health/reproductive.

## Data Availability

The datasets used and/or analysed during the current study available from the corresponding author on reasonable request (Melyane Gaia, mellyane@gmail.com).
